# Metallo-β-lactamase and AmpC genes in *Escherichia coli, Klebsiella pneumoniae*, and *Pseudomonas aeruginosa* isolates from abattoir and poultry origin in Nigeria

**DOI:** 10.1186/s12866-021-02179-1

**Published:** 2021-04-21

**Authors:** Chika Ejikeugwu, Okoro Nworie, Morteza Saki, Hussein O. M. Al-Dahmoshi, Noor S. K. Al-Khafaji, Chika Ezeador, Emmanuel Nwakaeze, Peter Eze, Eniola Oni, Chidiebere Obi, Ifeanyichukwu Iroha, Charles Esimone, Michael U. Adikwu

**Affiliations:** 1 Department of Applied Microbiology, Ebonyi State University, Abakaliki, Nigeria; 2 Department of Biological Sciences, Alex Ekwueme Federal University, Ndufu-Alike Ikwo, Ikwo, Nigeria; 3 Department of Microbiology, Faculty of Medicine, Ahvaz Jundishapur University of Medical Sciences, Ahvaz, Iran; 4 Biology Department, College of Science, University of Babylon, Hilla City, Babylon Province Iraq; 5 Department of Medical Microbiology & Parasitology, Nnamdi Azikiwe University, Awka, Nigeria; 6 Department of Environmental Health Science, Nnamdi Azikiwe University, Awka, Nigeria; 7 Department of Microbiology, Federal University of Agriculture, Abeokuta, Nigeria; 8 Department of Microbiology, Federal University, Birnin Kebbi, Nigeria; 9 Department of Pharmaceutical Microbiology and Biotechnology, Nnamdi Azikiwe University, Awka, Nigeria; 10 Department of Pharmaceutics, University of Nigeria, Nsukka, Nigeria

**Keywords:** AmpC, Antibiotic resistance, Gram-negative bacteria, Metallo-β-lactamase, Multidrug resistance, Nigeria

## Abstract

**Background:**

Gram-negative bacteria (GNB) including *Escherichia coli*, *Pseudomonas aeruginosa*, and *Klebsiella pneumoniae* represent the most relevant reservoir of resistance genes such as metallo-β-lactamase (MBL) and AmpC genes that give them the undue advantage to resist antimicrobial onslaught. This study aimed to investigate the occurrence of MBL (*bla*_IMP-1_, *bla*_IMP-2_, *bla*_VIM-1_, *bla*_VIM-2_) and AmpC (*bla*_FOX_, *bla*_DHA_, *bla*_CMY_, *bla*_ACC_) resistance genes in aforementioned GNB collected from abattoir and poultry sources in Nigeria.

**Results:**

In total, 370 isolates were collected from abattoir tables (*n* = 130), anal region of cows (*n* = 120), and the cloacae of poultry birds (*n* = 120). The test isolates showed high rate of resistance to cephalosporins and carbapenems. The MBLs were phenotypically detected in 22 *E. coli*, 22 *P. aeruginosa*, and 18 *K. pneumoniae* isolates using combined disc test (CDT). However, only 11 *E. coli*, 24 *P. aeruginosa*, and 18 *Klebsiella pneumoniae* isolates were phenotypically confirmed to be AmpC producers using cefoxitin-cloxacillin double disk synergy test (CC-DDST). MBL encoding genes (particularly the *bla*_IMP-1_ genes and *bla*_IMP-2_ genes) were detected by polymerase chain reaction (PCR) in 12 (54.6%) *E. coli*, 15 (83.3%) *K. pneumoniae*, and 16 (72.7%) *P. aeruginosa* isolates. AmpC genes (particularly the *bla*_CMY_ genes and *bla*_FOX_ genes) were found in a total of 5 (29.4%) *E. coli* isolates, 5 (27.8%) isolates of *K. pneumoniae*, and 10 (41.7%) isolates of *P. aeruginosa*.

**Conclusions:**

Our study showed the circulation of MBL and AmpC genes in GNB from abattoir and poultry origin in Nigeria. Adoption of regular control policies is necessary to reduce the spread of these species as soon as possible, especially in poultry and slaughterhouses.

## Background

There is plethora of reports about the prevalence of metallo-β-lactamase (MBL) and AmpC beta-lactamase-producing bacteria in the hospital environment, community, and food-producing animals [1–3]. The biggest challenge facing the healthcare sectors in developing countries such as Nigeria is the emergence and spread of multidrug-resistant (MDR) bacteria including *Escherichia coli*, *Pseudomonas aeruginosa*, and *Klebsiella pneumoniae* harboring aforesaid elements [4]. Measuring the prevalence of MDR bacteria coupled with rational use of available antibiotics and proper susceptibility testing is critical to reducing the possible risks associated with infections due to multidrug resistance [5, 6]. Both carbapenem-resistant *Enterobacteriaceae* and AmpC-producing Gram-negative bacteria (GNB) have emerged as important health issue that is of global health concern [7, 8]. This trend can adversely affect the World Health Organization (WHO) ‘One Health’ policy because of the ease with which GNB harboring multidrug resistance genes can zoonotically move from animals to humans and vice versa [9]. The AmpC β-lactamases are clinically important beta-lactamases that confer antimicrobial resistance to the narrow-spectrum, expanded-spectrum, and broad-spectrum cephalosporins, and β-lactamase inhibitors such as amoxicillin-clavulanic acid [10, 11]. On the other hand, MBLs are beta-lactamase enzymes which hydrolyze the carbapenems such as imipenem and render them ineffective for therapeutic purposes [2]. The presence of MBLs and AmpC enzymes in the pathogenic bacteria reduces the therapeutic effectiveness of antibiotics because of their multidrug resistance properties. The possible contamination of food and food-producing animals with MDR bacteria harboring MBLs and AmpC enzymes is perhaps a potential source for the wide dissemination of antibiotic resistance [12]. We hypothesized that *E. coli*, *P. aeruginosa*, and *K. pneumoniae* from abattoir origin harbor genetic factors that mediate the production of MBL and AmpC enzymes as important resistance phenotypes. This study aimed to investigate the occurrence rate of MBL and AmpC genes in GNB isolates collected from abattoir and poultry sources in Nigeria.

## Results

### Distribution of the bacterial isolates in the abattoir and poultry samples

The distribution of the isolated bacteria is shown in Table 1. A total of 456 non-duplicate isolates were identified as *E. coli*, *P. aeruginosa*, and *K. pneumoniae* in 370 samples collected. The *E. coli* with frequency of 36.8% (168/456) was the most prevalent species, followed by *P. aeruginosa* (*n* = 147; 32.2%), and *K. pneumoniae* (*n* = 141; 30.9%).

**Table 1:** Isolation rate of the *Escherichia coli*, *Pseudomonas aeruginosa*, and *Klebsiella pneumoniae* from different samples

Organism	Swabs from abattoir benches (***n*** = 165) n (%)	Cloacal swabs of poultry birds (***n*** = 148) n (%)	Anal swabs of cow (***n*** = 143) n (%)	Total (***n*** = 456) n (%)
*Escherichia coli*	69 (41.8)	51 (34.5)	48 (33.6)	168 (36.8)
*Pseudomonas aeruginosa*	56 (33.9)	48 (32.4)	43 (30.1)	147 (32.2)
*Klebsiella pneumoniae*	40 (24.2)	49 (33.1)	52 (36.4)	141 (30.9)

### Antimicrobial susceptibility

The antimicrobial susceptibility testing results are shown in Table 2. The most resistance rate was seen against cefotaxime (91.7%) followed by ceftriaxone (83.6%), aztreonam (83.1%), and ciprofloxacin (82.9%). The carbapenems (imipenem and meropenem) with the susceptibility rate of 46.7% were the most effective antibiotics. High levels of resistance were noted in the *E. coli* isolates that showed varying rates of resistance. The *E. coli* isolates showed reduced susceptibility to cephalosporins: ceftriaxone (*n* = 160; 95.2%), ceftazidime (*n* = 162; 96.4%) and cefotaxime (*n* = 165; 98.2%). The resistance pattern most commonly observed amongst the *K. pneumoniae* isolates was resistance to cefotaxime (96.5%), aztreonam (96.5%), ceftriaxone (89.4%), ciprofloxacin (86.5%), and ceftazidime (82.3%). The antibiotic resistance pattern of the organism confirmed that more than 50% of the *P. aeruginosa* isolates showed high-level of resistance to the carbapenems including imipenem (66.7%), ertapenem (61.2%) and meropenem (60.5%). Reduced susceptibility of the *P. aeruginosa* isolates was also observed in cephalosporins: cefoxitin (80.3%), cefotaxime (79.6%), ceftriaxone (64.6%), and ceftazidime (54.4%).

**Table 2:** Susceptibility test results of all isolates

Antibiotics (μg)	***Escherichia coli***		***Klebsiella pneumoniae***		***Pseudomonas aeruginosa***		Total	
	S n (%)	R n (%)	S n (%)	R n (%)	S n (%)	R n (%)	S n (%)	R n (%)
CRO (30)	8 (4.8)	160 (95.2)	15 (10.6)	126 (89.4)	52 (35.4)	95 (64.6)	75 (16.4)	381 (83.6)
FOX (30)	43 (25.6)	125 (74.4)	35 (24.8)	106 (75.2)	29 (19.7)	118 (80.3)	107 (23.5)	349 (76.5)
IPM (10)	81 (48.2)	87 (51.8)	83 (58.9)	58 (41.1)	49 (33.3)	98 (66.7)	213 (46.7)	243 (53.3)
CAZ (30)	6 (3.6)	162 (96.4)	25 (17.7)	116 (82.3)	67 (45.6)	80 (54.4)	98 (21.5)	358 (78.5)
ETP (10)	22 (13.1)	146 (86.9)	22 (15.6)	119 (84.4)	57 (38.8)	90 (61.2)	101 (22.1)	355 (77.9)
OFX (5)	49 (29.2)	119 (70.8)	49 (34.8)	92 (65.2)	54 (36.7)	93 (63.3)	152 (33.3)	304 (66.7)
GM (10)	73 (43.5)	95 (56.5)	55 (39.0)	86 (61.0)	65 (44.2)	82 (55.8)	193 (42.3)	263 (57.7)
AK (10)	59 (35.1)	109 (64.9)	67 (47.5)	74 (52.5)	53 (36.1)	94 (63.9)	179 (39.3)	277 (60.7)
CIP (5)	31 (18.5)	137 (81.5)	19 (13.5)	122 (86.5)	28 (19.0)	119 (81.0)	78 (17.1)	378 (82.9)
CTX (30)	3 (1.8)	165 (98.2)	5 (3.5)	136 (96.5)	30 (20.4)	117 (79.6)	38 (8.3)	418 (91.7)
MEM (10)	75 (44.6)	93 (55.4)	80 (56.7)	61 (43.3)	58 (39.5)	89 (60.5)	213 (46.7)	243 (53.3)
AMP (10)	50 (29.8)	118 (70.2)	43 (30.5)	98 (69.5)	28 (19.0)	119 (81.0)	121 (26.5)	335 (73.5)
ATM (30)	11 (6.5)	157 (93.5)	5 (3.5)	136 (96.5)	61 (41.5)	86 (58.5)	77 (16.9)	379 (83.1)

*S* Susceptible, *R* Resistant, *IPM* Imipenem, *MEM* Meropenem, *ETP* Ertapenem, *FOX* Cefoxitin, *CAZ* Ceftazidime, *AK* Amikacin, GM Gentamicin, *CTX* Cefotaxime, *CRO* Ceftriaxone, *CIP* Ciprofloxacin, *OFX* Ofloxacin, *AMP* Ampicillin, *ATM* Aztreonam

### Phenotypic characterization of MBL and AmpC enzyme production

The Table 3 shows the result of the phenotypic confirmation of MBL production in the tested bacteria. MBL producing bacteria was phenotypically detected in a total of 22 (13.1%) *E. coli* isolates. Among *K. pneumoniae* isolates, 6, 7, and 5 isolated from abattoir tables, cloacal swabs of poultry birds, and anal swab samples of cows were identified as MBL producers, respectively. In this study, *P. aeruginosa* isolates that produce MBL enzymes were phenotypically detected in abattoir samples (12.5%), cloacal swab samples (14.6%), and anal swab samples of cows (18.6%).

**Table 3:** Phenotypic occurrence of metallo-β-lactamase (MBL) in studied bacteria

Bacteria (n)	Source	Positive MBL n (%)
*Escherichia coli* (69)	Abattoir	8 (11.6)
*Escherichia coli* (51)	Poultry	7 (13.7)
*Escherichia coli* (48)	Anal swabs of cow	7 (14.6)
*Pseudomonas aeruginosa* (56)	Abattoir	7 (12.5)
*Pseudomonas aeruginosa* (48)	Poultry	7 (14.6)
*Pseudomonas aeruginosa* (43)	Anal swabs of cow	8 (18.6)
*Klebsiella pneumoniae* (40)	Abattoir	6 (15.0)
*Klebsiella pneumoniae* (49)	Poultry	7 (14.3)
*Klebsiella pneumoniae* (52)	Anal swabs of cow	5 (9.6)
Total (456)		62 (13.6)

The result of the AmpC enzyme production in the *E. coli, K. pneumoniae*, and *P. aeruginosa* isolates is shown in Table 4. AmpC enzyme was phenotypically detected in *E. coli* isolates from abattoir (8.7%), cloacal swab samples (9.8%), and anal swabs of cows (12.5%). On the other hand, 9 isolates of *P. aeruginosa* (16.1%) from abattoir tables and 7 isolates (14.6%) from poultry sources were confirmed as AmpC enzyme producers. It was also found that 8 isolates of *P. aeruginosa* (18.6%) from the anal swabs of cows were phenotypically confirmed as AmpC enzyme producers. AmpC enzyme production amongst the *K. pneumoniae* isolates was 15.0% in abattoir tables, 12.2% in poultry specimens and 11.5% in the anal swabs of cows. The MBL and AmpC positive *Klebsiella* species have MARI of 0.4 to 0.5, while the MBL and AmpC positive *E. coli* isolates have MARI of 0.4 to 0.6. The *P. aeruginosa* isolates that were positive for MBL and AmpC enzyme production had a MARI of 0.7 to 0.8. The MARI result showed that the MBL and AmpC positive *K. pneumoniae* and *E. coli* isolates were multiply resistant to more than four antibiotic classes while the *P. aeruginosa* isolates that were positive for MBL and AmpC enzyme production were multiply resistant to more than seven antibiotics.

**Table 4:** Phenotypic occurrence of AmpC producing bacteria

Organism (n)	Source	AmpC positive n (%)
*Escherichia coli* (69)	Abattoir	6 (8.7)
*Escherichia coli* (51)	Poultry	5 (9.8)
*Escherichia coli* (48)	Anal swabs of cow	6 (12.5)
*Pseudomonas aeruginosa* (56)	Abattoir	9 (16.1)
*Pseudomonas aeruginosa* (48)	Poultry	7 (14.6)
*Pseudomonas aeruginosa* (43)	Anal swabs of cow	8 (18.6)
*Klebsiella pneumoniae* (40)	Abattoir	6 (15.0)
*Klebsiella pneumoniae* (49)	Poultry	6 (12.2)
*Klebsiella pneumoniae* (52)	Anal swab of cow	6 (11.5)
Total (456)		59 (12.9)

### PCR detection of MBL and AmpC genes

The result of gene amplification of MBL and AmpC gene in the tested isolates is shown in Tables 5 and 6. Only the *bla*_IMP_ MBL gene family was detected by PCR in this study. The *bla*_VIM-1_ and *bla*_VIM-2_ MBL genes were not detected in the MBL phenotypes. Overall, the prevalence of *bla*_IMP-1_ gene in the MBL phenotypes was 36.4% (8/22), 50% (9/18), and 54.5% (12/22) for *E. coli, K. pneumoniae*, and *P. aeruginosa*, respectively. The *bla*_IMP-2_ gene was detected at the rate of 18.2% (4/22), 33.3% (6/18), and 18.2% (4/22) for *E. coli, K. pneumoniae*, and *P. aeruginosa* isolates, respectively. Also, only the *bla*_CMY_ and *bla*_FOX_ genes were detected in the studied isolates used in this study. Out of the 17 isolates of *E. coli*, only 3 (17.6%) and 2 (11.8%) isolates harbored the *bla*_CMY_ and *bla*_FOX_ genes, respectively. Four out of the 18 (22.2%) *K. pneumoniae* isolates harbored the *bla*_CMY_ gene, while only one isolate (5.6%) harbored the *bla*_FOX_ gene. The *bla*_CMY_ and *bla*_FOX_ genes were detected by PCR in 7 (29.2%) and 3 (12.5%) *P. aeruginosa* isolates, respectively. The co-existence of genes was not detected in any isolates. There was no association among the frequency of MBL and AmpC resistance genes with the studied isolates (*P*-value > 0.05) (Table 7).

**Table 5:** Total occurrence of MBL and AmpC genes in all 456 isolates

Genes	Occurrence n (%)
*bla*_IMP-1_	29 (6.4)
*bla*_IMP-2_	14 (3.1)
*bla*_VIM-1_	0 (0.0)
*bla*_VIM-2_	0 (0.0)
*bla*_CMY_	14 (3.1)
*bla*_FOX_	6 (1.3)
*bla*_DHA_	0 (0.0)
*bla*_ACC_	0 (0.0)

**Table 6:** Occurrence of MBL and AmpC genes variants among phenotypic MBL and AmpC positive *Escherichia coli, Klebsiella pneumoniae*, and *Pseudomonas aeruginosa*

**Genes**	***Escherichia coli*** **(*n*** **= 22)** **n (%)**	***Klebsiella pneumoniae*** **(*n*** **= 18)** **n (%)**	***Pseudomonas aeruginosa*** **(*n*** **= 22)** **n (%)**	**Total (*n*** **= 62)** **n (%)**
**MBL gene**				
*bla*_IMP-1_	8 (36.4)	9 (50)	12 (54.5)	29 (46.8)
*bla*_IMP-2_	4 (18.2)	6 (33.3)	4 (18.2)	14 (22.6)
Total	12 (54.6)	15 (83.3)	16 (72.7)	43 (69.4)
	***Escherichia coli*** **(*n*** **= 17)** **n (%)**	***Klebsiella pneumoniae*** **(*n*** **= 18)** **n (%)**	***Pseudomonas aeruginosa*** **(*n*** **= 24)** **n (%)**	**Total (*n*** **= 59)** **n (%)**
**AmpC gene**				
*bla*_CMY_	3 (17.6)	4 (22.2)	7 (29.2)	14 (23.7)
*bla*_FOX_	2 (11.8)	1 (5.6)	3 (12.5)	6 (10.2)
Total	5 (29.4)	5 (27.8)	10 (41.7)	20 (33.9)

**Table 7:** The MBL and AmpC gene presence association among the *E. coli*, *Klebsiella pneumoniae,* and *Pseudomonas aeruginosa*

Gene	***Escherichia coli*** (***n*** = 168) n (%)	***Klebsiella pneumoniae*** (***n*** = 141) n (%)	***Pseudomonas aeruginosa*** (***n*** = 147) n (%)	***P-***value
*bla*_IMP-1_	8 (5.0)	9 (6.0)	12 (8.2)	0.466901
*bla*_IMP-2_	4 (2.0)	6 (4.0)	4 (2.7)	0.608398
*bla*_VIM-1_	0	0	0	–
*bla*_VIM-2_	0	0	0	–
*bla*_CMY_	3 (1.8)	4 (2.8)	7 (4.8)	0.305612
*bla*_DHA_	0	0	0	–
*bla*_ACC_	0	0	0	–
*bla*_FOX_	2 (1.2)	1 (0.7)	3 (2.0)	0.602015

## Discussion

In this study, environmental samples from poultry, abattoir, and anal swabs of cows were bacteriologically analyzed for the isolation of *E. coli*, *K. pneumoniae*, and *P. aeruginosa* for multidrug resistance genes that encode MBL and AmpC enzymes production. The occurrence rates of 32.2 and 30.9% for *P. aeruginosa* and *K. pneumonia* in this study were higher than report by Savin et al. [13] from Germany who exhibited frequency rates of 5.1 and 10.8% for *P. aeruginosa* and *K. pneumoniae*, respectively. However, the occurrence rate of 36.8% for *E. coli* isolates was lower (39.4%) than that of Savin et al. Meanwhile, various previous studies in line with the current results, have shown a higher prevalence of *E. coli* in samples collected from environmental origin including poultry wastewater, and slaughter houses [13, 14]. The prevalence of GNB in slaughter houses and poultry farms is obvious due to the colonization of the gastrointestinal tract of animals with a variety of bacteria. However, since this high volume of bacteria may enter the human food chain and eventually lead to bacterial infections and the spread of antibiotic resistance, strict control policies should be implemented in the developing countries such as Nigeria to reduce the spread of MDR bacteria and increase the hygiene level of aforesaid environments [15, 16].

Abattoirs and poultry farms are good grounds for the evolution and spread of antibiotic resistant bacteria [17, 18]. The result of the AST in this study revealed a high level of resistance of *E. coli*, *K. pneumoniae*, and *P. aeruginosa* to more than 50% of the tested antibiotic panels including the cephalosporins, aminoglycosides, quinolones, and carbapenems. This is a possible indication of the stealth and exceptional emergence and spread of resistant GNB in the non-hospital environment. The results of this study were in parallel with the previous studies from Australia, China, and Switzerland that were suggestive of the ability of resistant GNB to cause a public health problem through the food chain, especially in climes where antibiotics are used irrationally in non-clinical practices [17–20]. In this study, the high resistance rates (> 70%) against third-generation cephalosporins (TGCs) were similar to previous report by Otokunefor et al. [21] from Nigeria who reported a resistance rate of ≥80% against TGCs family. Also, in accordance with the findings of this study, a high resistance rate against quinolones/fluoroquinolones was reported previously by a research from Romania [22]. However, the excessive high levels of resistance to quinolones (more than 60%) in comparison to a study from Iran by Talebiyan et al. [23] who reported a low resistance rate of 7.5% against ciprofloxacin is a cause for concern and makes the adoption of control measures clearer in Nigeria. The resistance rates against aminoglycosides family including amikacin and gentamicin were 60.7 and 57.7%, respectively, which were close to preceding report (58.4%) from Thailand and Cambodia [14]. In this study, 83.1% of isolates were resistant against aztreonam that was comparable to previous report by Elhariri et al. [24] who reported a 76.1% of resistance rate in *P. aeruginosa* isolates collected from camel meat samples at two major abattoirs in Egypt. Another finding of current study was the higher resistance rate of 73.5% against ampicillin in comparison to the research by Dsani et al. [25] from Ghana who reported resistance rate of 57% in *E. coli* isolates from raw meat.

One of the noteworthy results of this study was the high resistance of isolates to carbapenems. Although in this research the resistance of more than 50 % of isolates against carbapenems was very worrying, but still this category showed the highest efficacy among the studied antibiotics. In this study, the ertapenem with the susceptibility rate of 22.1% was the less effective carbapenem, while meropenem and imipenem were more effective. In the previous study from Palestinian territory, in contrary to this study, low resistance rates of 3 and 34% were reported against meropenem and imipenem, respectively [26]. One of the reasons for the presence of carbapenem-resistant bacteria in slaughter or poultry environments may be due to the use of broad-spectrum cephalosporins in these areas [3]. The exact link between the use of broad-spectrum cephalosporins and carbapenem resistance has not been fully investigated and disclosed. However, plasmids carrying both genes for resistance against TGCs (*bla*_ACC-1_) and carbapenems (*bla*_VIM-1_) have been found in bacteria isolated from farm animals [27].

In this study, we carried out CDT and CC-DDST to evaluate the presence of MBL and AmpC enzymes in the test GNB of *E. coli*, *K. pneumoniae*, and *P. aeruginosa* isolates. We also carried out PCR to determine the genes underpinning the multidrug resistance profile of the GNB. This current study was a follow-up to elucidate and give impetus to our previous studies on the emergence and spread of multidrug-resistant GNB in the community and the need to be on the lookout for these pathogens to forestall any disease outbreak due to them. Generally, most of the comprehensive data on MBL and AmpC producing GNB are from America, Europe, and partly Asia. There is still paucity of data on the prevalence of these pathogens in Africa and Nigeria. Today, there are various phenotypic methods for detection of different beta-lactamases, none of which have the sensitivity and specificity of molecular methods such as PCR and sequencing [28]. In this study, the total rates of phenotypic prevalence of studied MBLs and AmpC were 13.6% (62/456) and 12.9% (59/456), respectively. In the previous report by Ibadin et al. [29], lower rates of MBLs (8.1%) and AmpC (7.8%) were disclosed in GNB collected from clinical samples in Benin City, Nigeria. In another research from Italy the occurrence rate of 20% for MBLs and 16.7% for AmpC were stated in GNB collected from samples of fresh vegetables and ready-to-eat prepacked salads [30]. The difference in the prevalence rate of β-lactamases in these studies may be due to dissimilarities in the source of the sample collection and the methods used in the phenotypic detection of enzymes. Also, the results of phenotypic methods in the current experiment showed a higher prevalence of β-lactamase enzymes compared to PCR. The reason for this phenomenon can be due to the insensitivity and non-specificity of phenotypic methods and the existence of other genes such as *bla*_NDM_ that were not considered in this study due to severe financial constraints. Although the presence of genes such as *bla*_NDM_ and other MBLs has been rarely reported in animals, the investigation of these genes in the future studies will reveal more epidemiologic knowledge in our region [31].

The production of MBL and AmpC by GNB is worrisome because these pathogens have the exceptional ability to resist the antimicrobial onslaughts of carbapenems which are last-line antibiotics used in clinical medicine. The PCR method revealed the most incidences of MBL and AmpC genes in *P. aeruginosa* isolates followed by *K. pneumoniae* and *E. coli*. This survey revealed the total occurrence rates of 6.4% (29/456), 3.1% (14/456), 3.1% (14/456), and 1.3% (6/456) for *bla*_IMP-1,_ *bla*_IMP-2,_ *bla*_CMY,_ and *bla*_FOX,_ respectively. Unlike to the current results, a high frequency of *bla*_CMY_ (26.4%) was reported in companion animals in a study by Hong et al. [32] from South Korea, while *bla*_IMP,_ *bla*_VIM,_ and *bla*_FOX_ genes were not detected. In several earlier studies, the most reported AmpC gene was the *bla*_CMY_ gene [33]. As well, in this study, the *bla*_VIM_, *bla*_DHA_, and *bla*_ACC_ were not detected, while Hong et al. [32] reported the occurrence of *bla*_VIM_ and *bla*_DHA_ in their study. This showed that, epidemiologically, there are vast variances in different regions. These differences may be due to the dissimilarity in sample size, the method used for detection of various β-lactamase, and the source of sample collection.

### Limitation

In this study we do not sequence the PCR products due to the low financial sources. Another limitation was the lack of a modern technique such as multilocus sequence typing (MLST) and pulsed-field gel electrophoresis (PFGE) to determine the clonality of isolates.

## Conclusions

Antimicrobial resistance is fast becoming a significant global public health crisis that warrants concerted and sustainable measures to contain. And top amongst this is the effective surveillance, monitoring, detection, and prompt reporting of the development and spread of antimicrobial resistance in zoonotic and nosocomial pathogens, a practice which is still far-fetched in developing countries like Nigeria. The MBLs and AmpC genes detected amongst the isolates give GNB the undue advantage to resist the antimicrobial onslaught of antibiotics. The findings from this research are of major concern as scientific evidence from around the world continues to suggest that the overuse and inappropriate use of antibiotics in animal husbandry and poultry production contribute significantly to the emergence and spread of antibiotic resistant bacteria in the community. The epidemiological data from our work can serve as the basis for the development and establishment of antibiotic resistance surveillance unit’s across Nigeria’s states to assuage any disease outbreak due to these resistant strains.

## Methods

### Ethics

All methods in this study were carried out in accordance with the U.K. Animals (Scientific Procedures) Act, 1986 and associated guidelines, EU Directive 2010/63/EU for animal experiments. All experimental protocols of this study were approved by the Ethics Committee of Ebonyi State University, Nigeria, and all methods were carried out in compliance with the ARRIVE guidelines.

### Sample collection

Sample size determination for this study was determined by the Cochran’s formula. A total of 370 non-duplicate abattoir and poultry samples collected from abattoir benches (*n* = 130), anal region of cows (*n* = 120), and the cloacae of poultry birds, particularly broilers (*n* = 120) were recruited for this research from January to August 2019.

### Isolation of bacteria

The isolation, characterization, and identification of *E. coli, K. pneumoniae*, and *P. aeruginosa* from the abattoir and poultry samples was carried out using standard microbiology techniques including culture techniques, microscopy, biochemical testing, morphological and colonies features of the bacteria on selective culture media [34]. The isolates were bacteriologically, biochemically and microscopically confirmed to be *E. coli, Klebsiella pneumoniae*, and *P. aeruginosa*. *E. coli* ATCC 25922, *P. aeruginosa* ATCC 10145, and *K. pneumoniae* ATCC 700603 was used as control strains.

### Antimicrobial susceptibility testing (AST)

The modified Kirby-Bauer disk diffusion method was used for AST as per the guideline of Clinical Laboratory Standard Institute (CLSI) using single antibiotic discs: imipenem (IPM), meropenem (MEM), ertapenem (ETP), cefoxitin (FOX), ceftazidime (CAZ), amikacin (AK), gentamicin (GM), cefotaxime (CTX), ceftriaxone (CRO), ciprofloxacin (CIP), ofloxacin (OFX), ampicillin (AMP), aztreonam (ATM) on Mueller-Hinton (MH) agar plate (Oxoid, UK) according to a previous methodology [15, 35]. All test isolates were adjusted to 0.5 McFarland turbidity standard prior to culture and sensitivity study, and incubated at 37 °C for 18–24 h. The zone of inhibition was measured, recorded, and interpreted as susceptible (S), and resistant (R) using standard antibiotic breakpoints as stated by the CLSI [35].

### Multiple antibiotic resistance index (MARI)

The MARI indexes were determined for those bacterial isolates that showed positivity to MBL and AmpC enzymes production using a previously described methodology according to the following formula:

$$ \mathrm{MARI}=\frac{\mathrm{Total}\ \mathrm{number}\ \mathrm{of}\ \mathrm{antibiotics}\ \mathrm{that}\ \mathrm{each}\ \mathrm{isolate}\ \mathrm{was}\ \mathrm{resistant}\ \mathrm{against}\ \mathrm{them}}{\mathrm{Total}\ \mathrm{number}\ \mathrm{of}\ \mathrm{tested}\ \mathrm{antibiotics}\ \mathrm{for}\ \mathrm{each}\ \mathrm{islates}}\kern0.5em $$

[36].

### Phenotypic screening and detection of MBL and AmpC enzymes

*E. coli, Klebsiella pneumoniae*, and *P. aeruginosa* isolates that were found to show reduced susceptibility to the tested cephalosporins and carbapenems as per the antibiotic breakpoints recommended by CLSI for screening isolates for MBL and AmpC enzymes were phenotypically confirmed by previous methods for MBL and AmpC enzyme production using imipenem/imipenem + EDTA combined disc test (CDT) and the cefoxitin-cloxacillin double-disk synergy test (CC-DDST), respectively [37, 38].

### Isolation and preparation of DNA

DNA isolation and purification were carried out using the Zymo DNA miniprep kit (Epigenetics Company, USA) according to the manufacturer’s instruction. The extracted DNA samples were later stored at − 20 °C for the polymerase chain reaction (PCR) experiment.

### PCR detection of MBL and AmpC genes

The PCR amplification was performed in a thermal cycler (Lumex instrument, Canada) using specific primers (Table 8) synthesized and supplied by Inqaba Biotechnical Industries Ltd. (South Africa) as previously described [39, 40]. A final PCR mixture of 25 μl containing 0.2 μl of Taq polymerase enzyme (5 U/μL), 2.5 μl of 10X PCR buffer along with 2.5 μl MgCl2 (25 mM), 1 μl of 10 pM from each of the forward and reverse primers, 2.5 μl of dNTPs MIX (2 mM), 3 μl of DNA template (from the test isolates), and 12.3 μl of nuclease-free water were used for PCR amplification of MBL and AmpC genes. PCR amplification conditionfor the MBL genes was as follows: initial denaturation temperature at 95 °C for 2 min, followed by 25 cycles of DNA denaturation at 95 °C for 30 s, annealing (according to reference [19]) for 30 s, primer extension at 72 °C for 30 s, and a final extension step at 72 °C for 5 min. An MBL gene carrying *K. pneumoniae* from our previous study was used as a positive control [16]. The PCR amplification of AmpC genes was performed in following condition: initial denaturation temperature at 94 °C for 3 min, followed by 25 cycles of DNA denaturation at 94 °C for 30 s, annealing at 64 °C for 30 s, primer extension at 72 °C for 1 min, and a final extension at 72 °C for 7 min. An AmpC gene carrying *P. aeruginosa* from a previous study was used as a positive control [15].

**Table 8:** Primer sequences for amplification of MBL and AmpC genes

Gene targets	Primer sequence (5′ to 3′, as synthesized)	Expected amplicon size (bp)	Reference
**MBL genes**			
*bla*_IMP-1_	F1 (5′-ACCGCAGCAGAGTCTTTGCC-3′) R1 (5′-ACAACCAGTTTTGCCTTACC-3′)	587	[39]
*bla*_IMP-2_	F2 (5′-GTTTTATGTGTATGCTTCC-3′) R2 (5′-AGCCTGTTCCCATGTAC-3′)	678	[39]
*bla*_VIM-1_	F3 (5′-AGTGGTGAGTATCCGACAG-3′) R3 (5′-ATGAAAGTGCGTGGAGAC-3′)	261	[39]
*bla*_VIM-2_	F4 (5′-ATGTTCAAACTTTTGAGTAAG-3′) R4 (5′-CTACTCAACGACTGAGCG-3′)	801	[39]
**AmpC genes**			
*bla*_CMY_	F1 (5′-GCTGCTCAAGGAGCACAGGAT-3′)	520	[40]
	R1 (5′-CACATTGACATAGGTGTGGTG-3′)		
*bla*_DHA_	F1 (5′-AACTTTCACAGGTGTGCTGGGT-3′)	405	[40]
	R1 (5′-CCGTACGCATACTGGCTTTGC-3′)		
*bla*_ACC_	F1 (5′-AACAGCCTCAGCAGCCGGTTA-3′)	346	[40]
	R1 (5′-TTCGCC GCAATCATCCCTAGC-3′)		
*bla*_FOX_	F1 (5′-AACATG GGGTATCAGGGAGATG-3′)	190	[40]
	R1 (5′-CAAAGCGCGTAACCGGATTGG-3′)		

### Gel electrophoresis

Agarose gel electrophoresis of the PCR products was carried out using 1.5% agarose gel with ethidium bromide for 1 h at 80 V. The visual output of the amplicons was captured using an electrophoresis photography system (FotodyneFoto/Analyst Investigator, Fotodyne, Japan).

### Statistical analysis

Statistical evaluation was performed using SPSS, version 21.0 (Armonk, NY, USA). The analyzed data were presented as the descriptive frequencies. The comparison of variables was done by Fisher’s exact test and the *p*-value less than 0.05 was considered as a statistically significant association.

## Data Availability

All data generated or analyzed during this study are included here and are availablefrom the corresponding author on reasonable request.

## Abbreviations

CC-DDST: Cefoxitin-cloxacillin double disk synergy test
CDT: Combined disc test
CLSI: Clinical Laboratory Standard Institute
GNB: Gram-negative bacteria
MBL: Metallo-β-lactamase
MDR: Multidrug-resistant
PCR: Polymerase chain reaction
WHO: World Health Organization
